# A Co-Polymerizable Linker for the Covalent Attachment of Fibronectin Makes pHEMA Hydrogels Cell-Adhesive

**DOI:** 10.3390/gels8050258

**Published:** 2022-04-21

**Authors:** Laura Schumacher, Katharina Siemsen, Clement Appiah, Sunil Rajput, Anne Heitmann, Christine Selhuber-Unkel, Anne Staubitz

**Affiliations:** 1 Institute for Organic and Analytical Chemistry, University of Bremen, Leobener Straße 7, D-28359 Bremen, Germany; laura.schumacher1@ewetel.net (L.S.); appiah@uni-bremen.de (C.A.); heitmann@tutech.de (A.H.); 2 MAPEX Center for Materials and Processes, University of Bremen, Bibliothekstr. 1, D-28359 Bremen, Germany; 3 Biocompatible Nanomaterials, Institute for Materials Science, Kiel University, Kaiserstr. 2, D-24143 Kiel, Germany; katharina-siemsen@gmx.de; 4 Institute for Molecular Systems Engineering (IMSE), Heidelberg University, INF 253, D-69120 Heidelberg, Germany; sunil.rajput@uni-heidelberg.de; 5 Max Planck School Matter to Life, Jahnstraße 29, D-69120 Heidelberg, Germany

**Keywords:** polymer, pHEMA, bio-conjugation, hydrogel, biocompatibility

## Abstract

Hydrogels are attractive biomaterials because their chemical and mechanical properties can be tailored to mimic those of biological tissues. However, many hydrogels do not allow cell or protein attachment. Therefore, they are post-synthetically functionalized by adding functional groups for protein binding, which then allows cell adhesion in cell culture substrates. However, the degree of functionalization and covalent binding is difficult to analyze in these cases. Moreover, the density of the functional groups and the homogeneity of their distribution is hard to control. This work introduces another strategy for the biofunctionalization of hydrogels: we synthesized a polymerizable linker that serves as a direct junction between the polymeric structure and cell adhesion proteins. This maleimide-containing, polymerizable bio-linker was copolymerized with non-functionalized monomers to produce a bioactive hydrogel based on poly(2-hydroxyethyl methacrylate) (pHEMA). Therefore, the attachment site was only controlled by the polymerization process and was thus uniformly distributed throughout the hydrogel. In this way, the bio-conjugation by a protein-binding thiol-maleimide Michael-type reaction was possible in the entire hydrogel matrix. This approach enabled a straightforward and highly effective biofunctionalization of pHEMA with the adhesion protein fibronectin. The bioactivity of the materials was demonstrated by the successful adhesion of fibroblast cells.

## 1. Introduction

In nature, cells are surrounded by the extracellular matrix (ECM), which provides physical support and regulates cell behavior. Ubiquitous proteins in the ECM are, for instance, collagen, elastin, and the glycoproteins laminin and fibronectin. The latter mediate cell adhesion through integrins in the cell membrane [1,2,3]. Ideally, an artificial bioactive scaffold material should mimic such natural cell environments both chemically and mechanically [4,5].

Hydrogels are important biomaterials, mainly due to their mechanical properties, which are similar to those of soft tissue. Therefore, they can serve as materials for regenerative medicine [6,7,8] and particularly for controlling cellular functions by mechanotransduction [9,10]. Additionally, recent advances in the 3D structuring of hydrogels make them highly attractive for many biomaterial applications [11,12,13]. In general, hydrogels can be prepared and chemically modified by various methods. Many hydrogels are cross-linked polymers; the corresponding cross-linkers can be included in the monomer mixtures for free radical polymerization, but also physical crosslinking with ions exists [14]. By using different monomers or crosslinkers, and/or varying their ratios, the hydrogels’ properties can be adapted. For example, their stiffness and polymeric mesh size can be adjusted. Moreover, by the judicious choice of monomers and crosslinkers, the bio-degradability or biofunctionality of the hydrogel can be tailored [15,16]. A common method to biofunctionalize hydrogels is the attachment of functional entities, such as adhesion proteins, to the hydrogel surface [14]. Hydrogels serve as scaffold materials for in vitro cell experiments and tissue engineering [17,18,19,20,21,22,23], where a homogeneous functionalization of hydrogels is particularly relevant in the context of 3D cell culture [24,25].

There are a number of biocompatible hydrogels that are suitable for cell culture [26]. A technologically very important biocompatible hydrogel is pHEMA, which is often used in pharmaceutical applications [27,28] and for contact lenses [29]. It could also be a highly relevant material for biomedical applications because of its tunable mechanical properties [30]. Currently, although poly(2-hydroxyethyl methacrylate) (pHEMA) is biocompatible [14,31], these applications are limited because it does not allow the direct attachment of proteins intrinsically [17,32].

To create bioactive hydrogels of pHEMA, chemical modifications and additional biofunctionalizations can be employed. For example, atom transfer radical polymerization (ATRP) can be utilized to create a cell-adhesive surface with mono(2-methacryloyloxyethyl) phosphate [33] to promote cell adhesion. Moreover, surface structures of pHEMA brushes [34,35,36] or the creation of porous surface structures [37] can enhance cell adhesion. Although these methods lead to cell adhesive pHEMA, they require several post-processing steps after the synthesis of the hydrogel. Such post-processing steps do not easily translate into cell studies in 3D environments [38,39,40], where homogeneously functionalized 3D scaffolds are essential. For pHEMA, one study described methacrylated arginylglycylaspartic acid (RGD), which was copolymerized with HEMA monomers in order to achieve a thoroughly distribution of RGD in the hydrogel volume [41]. These RGD-pHEMAs allowed the attachment and invasion of human corneal epithelial cells.

A promising strategy to achieve biofunctionalization is to incorporate a bio-linker into the hydrogel precursor solution that can bind adhesion proteins such as fibronectin, collagen, or RGD to the hydrogel. This has already been successfully demonstrated for polyacrylamide (PAA) [42], by creating permanently crosslinked networks of PAA that sterically entrap high-molecular-weight linear polymers of PAA. The chemistry of the created systems allowed cell adhesion ligands such as collagen and fibronectin to be attached exclusively to the crosslinked elastic PAA network.

In this work, a polymerizable methacrylate-based bio-linker is presented, which can be co-polymerized with other methacrylates to form a hydrogel. The co-monomers in this case were (hydroxyethyl)methacrylate (HEMA) and ethylene glycol dimethacrylate (EGDMA). The bio-linker contains a maleimide group as the protein binding unit, which can bind to thiol groups in proteins such as fibronectin, or other proteins that contain cysteine groups [43,44,45,46]. Therefore, this bio-linker-containing hydrogel offers the possibility of performing in vitro experiments of cell adhesion by covalently binding fibronectin to the hydrogel. Furthermore, we also show that the hydrogel is biocompatible and that the bio-linker also influences the mechanical properties of the pHEMA hydrogel and can thus be used for tuning these properties as well.

## 2. Results and Discussion

### 2.1. Bio-Linker Synthesis

**Scheme 1:**
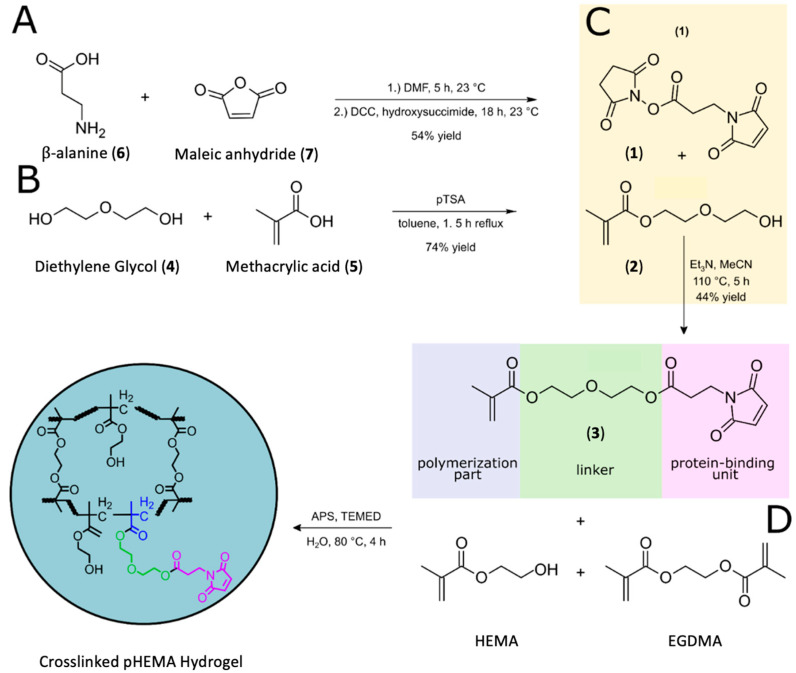
Synthesis of the bio-linker (**3**). (**A**) Synthesis of the protein binding unit; (**B**) functionalization of methacrylic acid; (**C**) synthesis of the bio-linker; (**D**) polymerization of the bio-linker.

To achieve a hydrogel suitable for protein binding, first the bio-linker (**3**) was synthesized from low-cost starting materials in a straightforward convergent 3-step synthesis (Scheme 1). The polymerization and the linker part were connected via an esterification, and the protein-binding unit was created from ß-alanine and maleic anhydride. The maleimide motif was used as a protein-binding unit for several reasons: the starting materials were cheap and readily available; moreover, the maleimide group is highly susceptible to react with thiol or amine groups. This improves the binding to adhesion molecules, as maleimides form stable thioether bonds between the maleimide’s double bond and thiol groups via a Michael-type reaction.

In the first step, maleic anhydride was reacted with the amino group of β-alanine in a one-pot procedure, giving the activated ester (**1**) with concomitant formation of the amine binding unit in a yield of 54% (Scheme 1A), similar to literature procedures for related compounds [47,48]. Building block 2 was prepared from diethylene glycol and methacrylic acid under high dilution conditions with an excess of diethylene glycol (DEG) to obtain the monosubstituted product in 71% yield (Scheme 1B). To obtain the novel bio-linker (**3**), in a final step, building blocks 1 and 2 were reacted in a nucleophilic addition reaction (Scheme 1A,B) with triethylamine as a base under reflux condition in acetonitrile (Scheme 1C). After purification, the bio-linker (**3**) was obtained with an overall yield of 44%.

### 2.2. Polymer Synthesis

The monomer units and bio-linker were synthesized according to the procedure described in the Supplementary Information (analysis shown in Figures S1–S4). To obtain reproducible gel formation and uniform hydrogels, it was necessary to degas the liquids employed (HEMA, EGDMA, and bio-linker (**3**)) before use. To achieve this, the liquids were placed in a glass vial sealed with a septum and were subsequently degassed. Since dissolved oxygen in the monomer reaction mixture suppresses the radical reaction, it lowers the polymerization efficiency as well as the crosslink density [49,50]. Removal of oxygen is therefore a necessary step to ensure a high degree of polymerization. Accordingly, by bubbling nitrogen through the solution, the reaction mixture was degassed for 30 min before the polymerization accelerator (TEMED) was added. If this step is omitted, areas of different stiffness or appearance will form in the hydrogel due to unevenly distributed monomers, and some parts of the polymer may detach after the final washing in water (Figure S5). Hence, sufficient degassing is an important step to ensure reproducibility of the synthetic method.

### 2.3. Rheology Measurements for Demonstration of Viscoelasticity of Hydrogels

**Figure 1:**
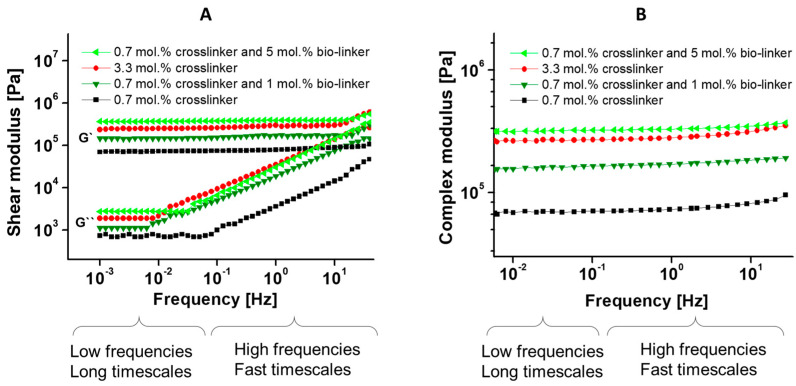
Repeated frequency dependence measurements of (**A**) storage (G′) and loss (G″) moduli and (**B**) complex modulus (G*) in Pa at 25 °C for the synthesized hydrogels with different percentages of cross-linker and bio-linker (**3**). As controls, the gels were also prepared without the bio-linker (**3**), with just the crosslinker, and with included bio-linker (**3**). The moduli were calculated from the mean value of three different samples each.

As the mechanical properties of a biomaterial strongly influence cells, particularly cell adhesion and stem cell differentiation [51,52,53], rheology measurements were carried out on the hydrogels to investigate their viscoelastic properties. Figure 1 shows the shear storage or elastic modulus (G′) and the loss or viscous modulus (G″) as a function of frequency for each hydrogel tested. The mechanical responses of all the investigated hydrogels at 25 °C were dominated by the G′ in the measured frequency range. This indicated that the elastic character of all the hydrogels dominated over their viscous character at 25 °C. The hydrogels showed a nearly frequency-independent response of G′ and G″ at low frequencies. At this frequency range, the hydrogels had a low modulus which varied little over the range of frequencies. At higher frequencies (fast timescales), the hydrogels showed more variation in the modulus, rising steeply with increasing frequency. The steep rise in modulus at high frequencies is due to low water content present in the hydrogels as a result of expulsion of water from the hydrogel network structure [53]. Complementary stress–strain curves characterizing the mechanical properties of the pHEMA hydrogels are shown in the (Supplementary Information Figure S6).

In the next step, the complex shear modulus, G*, which combines the storage modulus (elastic part) and the loss modulus (viscous part), was plotted against the frequency (ω); as shown in Equation (1).

(1) $$G^{*}=\sqrt{G\prime {\left(\omega \right)}^{2}+G\prime \prime {\left(\omega \right)}^{2}},$$

This graph was used to analyze the stiffness of the hydrogels. The higher the complex shear modulus, the stiffer the hydrogel. The resultant graph (Figure 1B) shows that an increase in the mol.% of the bio-linker leads to an increase in the complex shear modulus, which in turn indicates an increased elasticity of the hydrogel. The complex moduli were in the range of 58–99 kPa for pHEMA samples with 0.7 mol.% (1 wt.%) crosslinker, and 259–349 kPa with 3.3 mol.% (5 wt.%) crosslinker. With the addition of 1 mol.% (2.5 wt.%) bio-linker to the pHEMA with 0.7 mol.% (1 wt.%) crosslinker, the complex moduli nearly doubled (153–192 kPa) and were about four times higher for 5 mol.% (12.5 wt.%) bio-linker addition (316–352 kPa). This suggest that the samples with bio-linker (**3**) show an increased elasticity than the samples without bio-linker (**3**).

Shapiro, Oyen, and Peppas, et al. [54,55] showed that the mechanical properties of hydrogels depend on their polymeric structure and the hydrated state they can reach. Presumably, this effect also influenced the mechanical properties of the hydrogels in this study. Furthermore, the polymeric structure itself depends on the monomer conversion and the precursors used in the polymerization. The influence of the monomer-to-crosslinker concentration and the bio-linker (**3**) concentration towards the storage, loss, and complex moduli of the material was investigated using rheometer measurements. Our results on the storage, loss, and complex moduli showed increases according to the stoichiometric of the monomer-to-crosslinker and bio-linker (**3**) concentration. Our results from this study are also consistent with the literature on pHEMA-based hydrogels in terms of the frequency and crosslinker/bio-linker (**3**) concentration dependence [53,56,57,58,59].

### 2.4. MTT Assays for Assesment of Biocompatibility of Hydrogels

**Figure 2:**
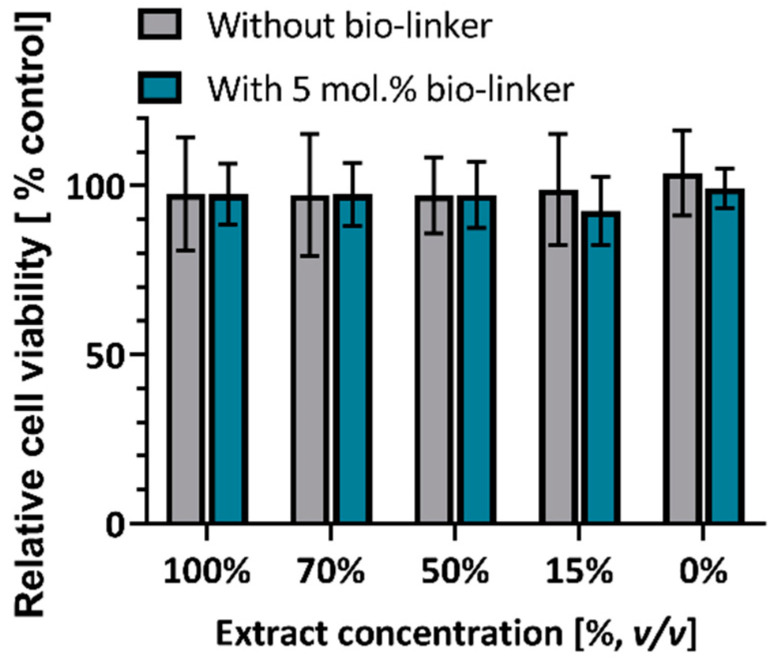
Results of the in vitro MTT cytotoxicity tests presented with mean value and standard deviation (at least three repeats) for different extract concentrations from 100% *v*/*v* to 0% *v*/*v* of the tested material. The tested materials were pHEMA samples with 0.7 mol.% EGDMA, without bio-linker (grey), and with 5 mol.% bio-linker (blue). Cell viabilities were above 90% for all tested samples.

To apply the synthesized pHEMA materials in a biomedical context, the samples need to be biocompatible. Therefore, an MTT extraction assay was used to investigate the biocompatibility of the synthesized materials. All tested samples were biocompatible for all extract concentrations with a cell viability above 90% (Figure 2) [60,61], demonstrating the suitability of the bio-linker-containing hydrogel for cell culture. This suggests that our strategy for the formation of the hydrogel is also suitable for many other biological applications where pHEMA has been used [14,62,63,64].

### 2.5. Cell Adhesion on pHEMA Modified with Bio-Linker (**3**)

**Figure 3:**
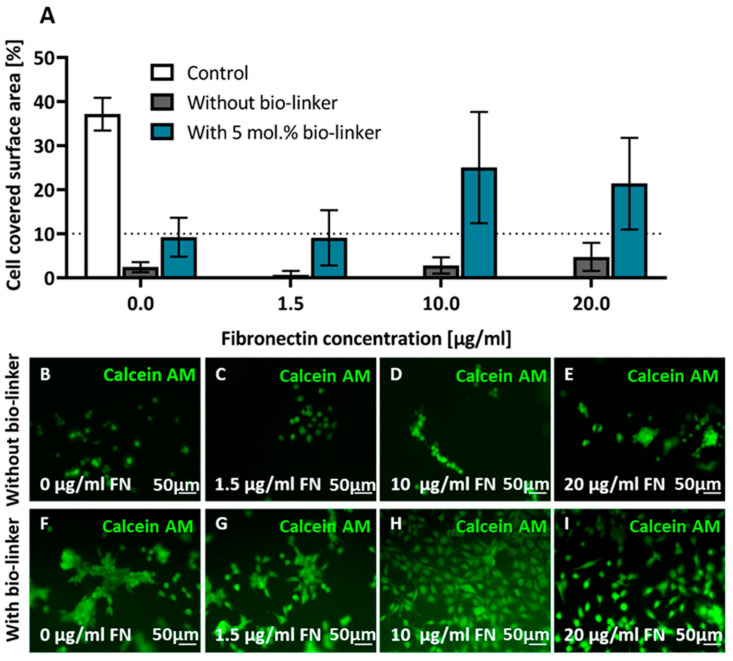
(**A**) Cell surface coverage determined from calcein AM fluorescence of fibroblast cells after 24 h incubation on pHEMA hydrogels (1 wt.% crosslinker, without (grey) and with bio-linker (**3**) (blue)) as a function of fibronectin concentration, compared to a control sample (petri dish). Increasing fibronectin concentration leads to an increase of cell coverage (data: mean ± standard deviation). Control: 12-well plates. (**B**–**E**) Representative images of calcein-AM-stained cells on pHEMA surfaces without bio-linker 3, (**F**–**I**) with bio-linker. Cell adhesion is significantly improved on hydrogels containing the bio-linker. Scale bar: 50 µm.

To investigate if the bio-linker improves fibronectin binding and thus cell adhesion in the volume of the pHEMA material, fibroblast cells were grown for 24 h on the surface of hydrogel slices (Figure 3) and then stained with calcein AM, which only stains live cells (Figure 3B). Only samples that contained the bio-linker (**3**) and fibronectin led to a significant cell surface coverage. In addition, cell surface coverage increased with increasing fibronectin concentration. As shown in Figure 3A, the highest cell coverage in pHEMA samples was achieved in samples containing the bio-linker (**3**) and at least 10 µg/mL fibronectin. While cells had a roundish shape and were mostly clustered in hydrogels without the bio-linker (**3**), cells spread in samples that contained the bio-linker and had a fibronectin concentration above or equal 10 µg/mL. Thus, cells were more evenly distributed in the latter samples, and cluster formation was reduced.

pHEMA-based hydrogels are known for the low numbers of adhering cells to unmodified pHEMA [55], but also for their high biocompatibility. The bio-linker (**3**) significantly improved cell adhesion without additional post-procedures compared to other studies [35,37,65], demonstrating the potential of our synthesis strategy to make pHEMA a valuable material for achieving cell adhesion, even in interior sections of the hydrogel. We also observed that the fibronectin concentration had an impact on cell adhesion, which is in agreement with previous research [65].

## 3. Conclusions

In conclusion, a new bio-linker (**3**) was developed for the synthesis of biofunctionalizable pHEMA hydrogels. The synthesis of the bio-linker (**3**) was very efficient, with an overall yield of 44% in three steps. The bio-linker (**3**) enables the fabrication of ready-to-use hydrogel in a two simple steps. This is relevant for high through-put biofunctionalization options for cell investigations. As a basis for this study, the highly medically relevant hydrogel pHEMA was selected, but in principle, this strategy can be adapted to other matrix materials that are synthetized by radical polymerization. We could demonstrate that the bio-linker (**3**) was fully biocompatible and led to significantly increased cell adhesion. Our study validated the potential of the bio-linker (**3**) with a maleimide motif in future biomedical applications, as it facilitates biofunctionalization within the whole hydrogel matrix without corrupting the network integrity, which is particularly relevant in 3D materials.

## 4. Materials and Methods

All commercially available reagents and solvents for the synthesis were used as received unless noted otherwise. The solvents for the syntheses were degassed with nitrogen prior to use. 3-Maleimidopropionic acid *N*-hydroxysuccinimide ester (**1**) and 2-(2-hydroxyethoxy)ethyl methacrylate (**2**) were prepared according to literature procedures [47,48]. Unless otherwise specified, all polymerization reactions were performed in glass vials under a nitrogen atmosphere by bubbling nitrogen gas through the solution for 30 min.

Column chromatography was performed on silica gel 60 (Merck, Darmstadt, Germany) with a pore size of 15–40 µm. Analytical thin layer chromatography (TLC) was performed on silica gel 60 F254 plates from Merck. Visualization was achieved by a KMnO_4_ (0.0475 M) containing staining solution.

^1^ H NMR spectra and ^13^ C{^1^ H} NMR spectra were recorded on a on a Bruker DRX-600 (600 MHz) spectrometer at 298 K. Chemical shifts are referenced to the residual proton of the deuterated solvent (CDCl_3_: δ (^1^ H) = 7.26 ppm, δ (^13^ C{^1^ H}) = 77.2 ppm). All chemical δ shifts are given in parts per million (ppm) and all coupling constants J in Hz. Full assignment of the peaks was achieved with the aid of 2D NMR techniques (^1^ H/^1^ H COSY and ^1^ H/^13^ C HSQC).

Electron impact (EI) ionization mass spectra were obtained on the double focusing mass spectrometer MAT 95+ or MAT 8200 from Finnigan Mat (Bremen, Germany). Samples were measured by direct or indirect inlet method with a source temperature of 200 °C. The ionization energy of the electron impact ionization was 70 eV. All signals were reported with the quotient from mass to charge *m*/*z*. High resolution mass spectrometric (HRMS) measurements were performed in the positive ion mode using a JEOL-Accu TOF 4GGCV EI mass spectrometer or on the double focusing mass spectrometer MAT 95+ or Mat 8200 from Finnigan Mat. Electron ionization (EI) was performed using an ionization potential of 70 eV. Atmospheric pressure chemical ionization (APCI) experiments were performed on a Bruker Impact II (Bruker Daltonics, Billerica, MA, USA). The calculated isotopic distribution for each ion was in agreement with experimental values.

IR spectra were recorded on a Nicolet Thermo iS10 Scientific IR spectrometer (Thermo Fisher Scientific, Schwerte, Germany) with a diamond-ATR-unit. The resolution was 4 cm^−1^. Relative intensities of the IR bands were described as s = strong, m = medium, or w = weak.

### 4.1. pHEMA Polymer Synthesis

The co-polymerization reaction of the hydrogels was achieved with different concentrations of the monomers HEMA, EGDMA, and bio-linker (**3**) in a small reaction ampoule (glass vial, 10 mL). HEMA (500 mg, 770 µmol), EGDMA (1–5 wt.%), bio-linker (**3**) (2.5–12.5 wt.%), ammonium persulfate (APS) (0.1 wt.%), and deionized water (0.50 mL) were added to the vial, which was then capped with a rubber septum and degassed by bubbling nitrogen gas through the solution for 30 min. The mixture was placed in an ultrasonic bath for 5 min and subsequently heated at 80 °C for another 5 min in a water bath. Tetramethylethylendiamine (TEMED) (0.15 wt.%) was added using a micro syringe, and the mixture was placed in an ultrasonic bath for 1 min to ensure uniform distribution of the TEMED in the reaction medium. The reaction ampoule was transferred to a water bath at 80 °C and kept there for 4 h, allowing hydrogel formation. The obtained hydrogels were removed and placed in deionized water (20 mL) for at least 3 days to remove unreacted excess monomers. The water was exchanged every 24 h.

### 4.2. Rheology

Rheology experiments were carried out on a Kinexus Pro+ MAL1196589 rheometer (Malvern, Herrenberg, Germany). A parallel plate system with a plate diameter of 20 mm was used. The sample temperature was controlled by passive heating/cooling exchanger under an air atmosphere. The lower plate was connected to the passive heat exchanger and controlled the sample temperature. To prevent water from evaporating entirely from the hydrogel structure and to maintain constant measurement conditions, a humidity cover (solvent cooling trap system) was placed over the hydrogel. All the measurements were subsequently performed in a dynamic mode with a frequency range of 0.001–100 Hz and a small strain amplitude of 0.06%, which was obtained from the amplitude sweep measurements. The samples to be measured were cut into 3 to 4 small pieces of approximately 1 cm^2^ and were measured separately. Samples were equilibrated for approximately 30 min between the two plates at constant measuring temperature (25 °C) before beginning the measurements. The elastic and viscous moduli for each sample were calculated from the mean value of at least three hydrogel specimen measurements.

### 4.3. Cell Culture

For all experiments, wild-type rat embryonic fibroblasts (REF 52 wt) were cultivated at 37 °C and a 5% CO_2_ atmosphere in an Heracell VIOS160i incubator (Thermo Fisher Scientific, Schwerte, Germany). The cultivation medium consisted of Dulbecco’s Modified Eagle’s Medium (DMEM; Merck, Darmstadt, Germany) with 10% foetal bovine serum (FBS; Biochrom, Berlin, Germany) and 1% penicillin-streptomycin (penstrep; Biochrom, Berlin, Germany). During cultivation, cells were grown up to 70–80% confluency. A control sample of the cells grown for 24 h in a 12-well plate is shown in Figure S7. For cell detachment, 0.04 mL accutase (Merck, Darmstadt, Germany) per cm² was added to the cell culture flask with subsequent incubation for 5–10 min. Detached cells were suspended in fresh cell medium, centrifuged using a Heraeus Megafuge (ThermoScientific, Germany) at 8800× *g*, for 4 min, and the cell pellet was afterwards resuspended in fresh medium. Cells were counted with a cell scepter (Merck, Darmstadt, Germany) and seeded in the desired concentration. For cell adhesion assays, 50,000 cells per sample, and for MTT assays, 10,000 cells per well of a 96-well plate (Sarstedt, Nümbrecht, Germany) were seeded.

### 4.4. MTT Assay

The synthesized bio-linker-containing hydrogels were analyzed using an MTT (3-(4,5-dimethylthiazol-2-yl)-2,5-diphenyltetrazolium chloride; Sigma Aldrich, Steinheim am Albuch, Germany) assay, according to ISO 10993, to prove their biocompatibility. The sample extracts were obtained as follows: the hydrogels were cut into 10 × 2.5 × 2.5 mm pieces, sterilized with 70% EtOH for 15 min, and placed in cell medium for 72 h. In 96-well plates, 10,000 cells per well were seeded in 100 µL cell medium and incubated for 24 h. Eventually, the medium was removed from the semi-confluent cell layer and washed with PBS (1 × 2 mL) before sample extracts were added at different volume concentration in DMEM (100%, 70%, 50%, 15%, 0% *v*/*v*). 100% *v*/*v* DMEM served as negative control, and 20% *v*/*v* dimethyl sulfoxide (DMSO; Sigma Aldrich, Steinheim am Albuch, Germany) as positive control. The cells were incubated for 24 h, then the solution was exchanged to 50 µL MTT solution (1.00 mg/mL MEM Earle’s; Gibco, NY, USA) and incubated for 2 h. Within this time, the MTT is metabolized by the cell and converted to purple formazan crystals. This solution was removed and replaced by 100 µL isopropanol (Walter, Germany) to dissolve the resulting formazan crystals. UV/Vis absorption of the assay was conducted at a wavelength of 570 nm in a EPOCH|^2^ microplate reader (BioTek Instruments Inc., Switzerland). The cell viability in percent was calculated by a standard equation after subtracting the optical density (OD) mean value of the empty 96 wells from each OD of the treated cells:

(2) $$\mathrm{Viability}\left[\%\right]=\frac{{\mathrm{OD}}_{\mathrm{sample}}\cdot 100}{{\mathrm{OD}}_{\mathrm{control}}}$$

Here, OD_sample_ is the optical density of the formed formazan metabolized from cells in contact with the extract of the test material, and OD_control_ is the optical density of the formazan formed from the cells treated with negative control medium.

A material is considered to be cytotoxic if its 100% *v*/*v* extract has a cell viability of less than 70% [60] For all materials tested here, four independent experiments were conducted, each with at least three replicates.

### 4.5. Hydrogel-Functionalisation with Fibronectin

**Figure 4:**
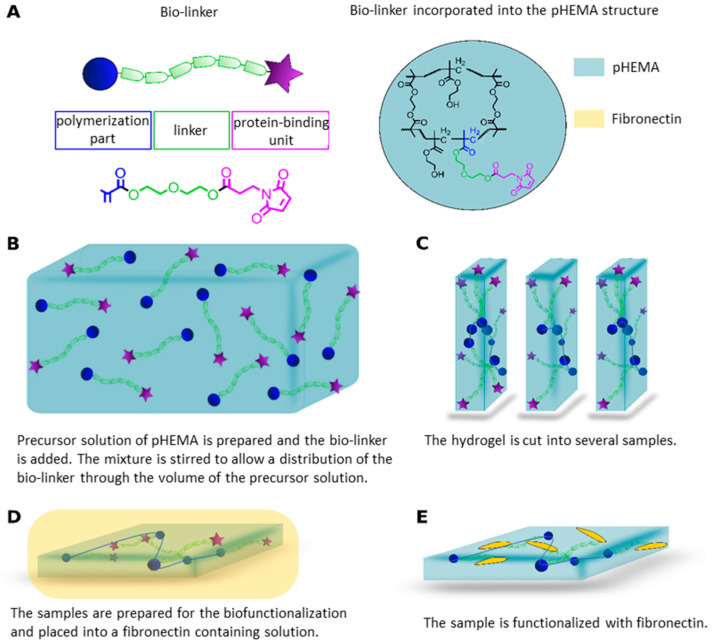
Schematic overview of the functionalization of pHEMA hydrogels with fibronectin. (**A**) Structure of the bio-linker (**3**): chemical structure of pHEMA with the incorporated bio-linker. (**B**,**C**) Hydrogel formation and sample preparation. (**D**) Functionalization with fibronectin (in yellow). (**E**) Fully functionalized material.

For the biofunctionalization with fibronectin (FN; Advanced BioMatrix, CA, USA), the samples were sterilized with EtOH (70% *v*/*v*) for 15 min and rinsed with 4-(2-hydroxyethyl)-1-piperazineethanesulfonic acid (HEPES, 50 mM, pH 8.5; Sigma Aldrich, Steinheim am Albuch, Germany) three times. Afterwards 3 µg, 20 µg, or 40 µg FN in 2 mL double distilled water was added, and the sample was stored at 4 °C for 24 h. Subsequently, the samples were washed with phosphate buffered saline (PBS; Sigma Aldrich, Steinheim am Albuch, Germany). A schematic overview is shown in Figure 4.

### 4.6. Cell Adhesion Assay

The influence of the fibronectin-functionalized pHEMA polymer was tested and evaluated with fibroblast cell adhesion assays. Both the cell surface coverage and cell morphology were determined by fluorescently stained cells. 50,000 cells in FlouroBrite medium (Gibco, USA) with 10% fetal bovine serum (FBS; Biochrom, Berlin, Germany) and 1% penicillin-streptomycin (Biochrom, Berlin, Germany) were cultured in the fibronectin-functionalized pHEMA samples for 24 h in an incubator at 37 °C. After 24 h, the cells were stained with calcein AM (live cell marker; BD Bioscience, Heidelberg, Germany) propidium iodide (dead cell marker; Thermo Fischer Scientific, Germany) as well as with Hoechst (nuclear marker; Thermo Fischer Scientific, Germany). Each fluorescent dye was prepared in a ratio of 1:1000 in FlouroBrite medium. The staining solution was then added to the samples containing the cells and placed in the incubator for 20 min. Afterwards, they were placed in the dark at room temperature for 10 min. The samples and cells were then washed three times with PBS to remove all excess dye from the samples and wells. Later, the samples were placed in a new petri dish with FlouroBrite medium for imaging.

### 4.7. Cell Imaging

Cell imaging was carried out using fluorescence microscopy, phase contrast microscopy, and bright field microscopy. A BX 43 microscope using the IC capture software (Olympus, Hamburg, Germany) with reflection and fluorescence microscopy using different objectives (10× UPLanFLN, 20× LcACH N; Olympus, Hamburg, Germany) was used to image the pHEMA samples. For the controls, i.e., cells on 6-well plates (Sarstedt, Germany), an inverted microscope (IX81) and CellSens software (Olympus, Hamburg, Germany); objectives: 10× UPLanFLN, 20× LcACH N (Olympus, Hamburg, Germany) was used. For each position, images in reflection and fluorescence mode were recorded. The imaging was conducted in FlouroBrite medium (Gibco, Germany) to limit background fluorescence.

### 4.8. Image/Data Analysis

For the analysis of the MTT assay photometer data, cell viability was calculated in the MTT assay section. The images recorded in cell adhesion assays were analysed with ImageJ [66]. For quantifying the surface area covered by cells, the ‘Analyze particles’ function in ImageJ was used (including a calculation of the mean and standard deviation).

## Data Availability

Not applicable.

## Supplementary Materials

The following supporting information can be downloaded at: https://www.mdpi.com/article/10.3390/gels8050258/s1, Table S1: List of Abbreviation; Table S2: List of suppliers and purity of the used solvents; Table S3: List of suppliers and purity of the used chemicals; Table S4: List of suppliers and purity of the used chemicals for cell cultivation and staining; Figure S1: Comparison of 1H NMR spectra of the starting materials (3-maleimidopropionic acid N-hydroxysuccinimide ester and 2-(2-hydroxyethoxy)ethyl methacrylate) and the bio-linker (3-maleimidopropionic acid diethyleneglycole methacrylate); Figure S2: HMBC spectra of the bio-linker (3-maleimidopropionic acid diethyleneglycole methacrylate). The coupling between C9 and H8 is indicated with an arrow and marked in the spectrum, to proof the success of the esterification reaction; Figure S3: ^13^ C{^1^ H} NMR spectrum of the starting material (3-maleimidopropionic acid *N*-hydroxysuccinimide ester); Figure S4: ATR-IR spectrum of the bio-linker (3-maleimidopropionic acid diethyleneglycole methacrylate); Figure S5: (**a**) hydrogels produced under oxygen atmosphere at 80 °C (**b**) hydrogels produced under nitrogen atmosphere at 80 °C; Figure S6: Stress-strain curves of the synthesized hydrogels with different percentages of cross-linker and bio-linker; Figure S7: Control of Ref 52 wt after 24 h of incubation on a 12-well plate (Sarstedt).

## References

[1] Frantz C., Stewart K.M., Weaver V.M. (2010). The extracellular matrix at a glance. J. Cell Sci..

[2] Bershadsky A., Chausovsky A., Becker E., Lyubimova A., Geiger B. (1996). Involvement of microtubules in the control of adhesion-dependent signal transduction. Curr. Biol..

[3] Ahearne M. (2014). Introduction to cell—hydrogel mechanosensing. Interface Focus.

[4] Ruprecht V., Monzo P., Ravasio A., Yue Z., Makhija E., Strale P.O., Gauthier N., Shivashankar V.G., Studer V., Albiges-Rizo C. (2017). How cells respond to environmental cues—Insights from bio-functionalized substrates. J. Cell Sci..

[5] Ross A.M., Jiang Z., Bastmeyer M., Lahann J. (2012). Physical Aspects of Cell Culture Substrates: Topography, Roughness, and Elasticity. Small.

[6] Huebsch N., Lippens E., Lee K., Mehta M., Koshy S.T., Darnell M.C., Desai R.M., Madl C.M., Xu M., Zhao X. (2015). Matrix elasticity of void-forming hydrogels controls transplanted-stem-cell-mediated bone formation. Nat. Mater..

[7] Slaughter V.B., Khurshid S.S., Fisher O.Z., Khademhosseini A., Peppas N.A. (2009). Hydrogels in regenerative medicine. Adv. Mater..

[8] Patel J.M., Loebel C., Saleh K.S., Wise B.C., Bonnevie E.D., Miller L.M., Carey J.L., Burdick J.A., Mauck R.L. (2021). Tissue Engineering: Stabilization of Damaged Articular Cartilage with Hydrogel-mediated Reinforcement and Sealing. Adv. Healthc. Mater..

[9] Lee-Thedieck C., Rauch N., Fiammengo R., Klein G., Spatz J.P. (2012). Impact of substrate elasticity on human hematopoietic stem and progenitor cell adhesion and motility. J. Cell Sci..

[10] Hadden W.J., Young J.L., Holle A.W., McFetridge M.L., Kim D.Y., Wijesinghe P., Taylor-Weiner H., Wen J.H., Lee A.R., Bieback K. (2017). Stem cell migration and mechanotransduction on linear stiffness gradient hydrogels. Proc. Natl. Acad. Sci. USA.

[11] Jungst T., Smolan W., Schacht K., Scheibel T., Groll J. (2016). Strategies and Molecular Design Criteria for 3D Printable Hydrogels. Chem. Rev..

[12] Gutekunst S.B., Siemsen K., Huth S., Möhring A., Hesseler B., Timmermann M., Paulowicz I., Mishra Y.K., Siebert L., Adelung R. (2019). 3D Hydrogels Containing Interconnected Microchannels of Subcellular Size for Capturing Human Pathogenic Acanthamoeba Castellanii. ACS Biomater. Sci. Eng..

[13] Mendes B.B., Daly A.C., Reis R.L., Domingues R.M.A., Gomes M.E., Burdick J.A. (2021). Injectable Hyaluronic Acid and Platelet Lysate-derived Granular Hydrogels for Biomedical Applications. Acta Biomater..

[14] Passos M.F., Dias D.R.C., Bastos G.N.T., Jardini A.L., Benatti A.C.B., Dias C.G.B.T., Maciel Filho R. (2016). pHEMA hydrogels: Synthesis, kinetics and in vitro tests. J. Therm. Anal. Calorim..

[15] Atzet S., Curtin S., Trinh P., Bryant S., Ratner B. (2008). Degradable poly(2-hydroxyethyl methacrylate)-co-polycaprolactone hydrogels for tissue engineering scaffolds. Biomacromolecules.

[16] Michel S.S.E., Dutertre F., Denbow M., Galan M.C., Briscoe W. (2019). Facile Synthesis of Chitosan-Based Hydrogels and Microgels through Thiol-Ene Photoclick Crosslinking. ACS Appl. Bio Mater..

[17] Klebe R.J., Bentley K.L., Schoen R.C. (1981). Adhesive substrates for fibronectin. J. Cell. Physiol..

[18] Engler A.J., Sen S., Sweeney H.L., Discher D.E. (2006). Matrix elasticity directs stem cell lineage specification. Cell.

[19] Hersel U., Dahmen C., Kessler H. (2003). RGD modified polymers: Biomaterials for stimulated cell adhesion and beyond. Biomaterials.

[20] Ribeiro A.J.S., Denisin A.K., Wilson R.E., Pruitt B.L. (2016). For whom the cells pull: Hydrogel and micropost devices for measuring traction forces. Methods.

[21] Naahidi S., Jafari M., Logan M., Wang Y., Yuan Y., Bae H., Dixon B., Chen P. (2017). Biocompatibility of hydrogel-based scaffolds for tissue engineering applications. Biotechnol. Adv..

[22] Michel S.S.E., Rogers S.E., Briscoe W., Galan M.C. (2020). Tuneable Thiol-Ene Photo Crosslinked Chitosan-Based Hydrogels for Biomedical Applications. ACS Appl. Bio Mater..

[23] Qazi T.H., Burdick J.A. (2021). Granular Hydrogels for Endogenous Tissue Repair. Biomater. Biosyst..

[24] Duarte C.D.F., Lindsay C.D., Roth J.G., LeSavage B.L., Seymour A.J., Krajina B.A., Ribeiro R., Costa P.F., Blaeser A., Heilshorn S.C. (2020). Bioprinting Cell- and Spheroid-Laden Protein-Engineered Hydrogels as Tissue-on-Chip Platforms. Front. Bioeng. Biotechnol..

[25] Siemsen K., Rajput S., Rasch F., Taheri F., Adelung R., Lammerding J., Selhuber-Unkel C. (2021). Tunable 3D Hydrogel Microchannel Networks to Study Confined Mammalian Cell Migration. Adv. Healthc. Mater..

[26] Caliari S.R., Burdick J.A. (2016). A practical guide to hydrogels for cell culture. Nat. Methods.

[27] Ayhan H., Ayhan F. (2018). Water based PHEMA hydrogels for controlled drug delivery. Turk Biyokim. Derg..

[28] Daly A.C., Riley L.A., Segura T., Burdick J.A. (2020). Hydrogel Microparticles for Biomedical Applications. Nat. Rev. Mater..

[29] Chatterjee S., Upadhyay P., Mishra M., Srividya M., Akshara M.R., Kamali N., Zaidi Z.S., Iqbal S.F., Misra S.K. (2020). Advances in chemistry and composition of soft materials for drug releasing contact lenses. RSC Adv..

[30] Guiseppi-Elie A., Dong C., Dinu C.Z. (2012). Crosslink density of a biomimetic poly(HEMA)-based hydrogel influences growth and proliferation of attachment dependent RMS 13 cells. J. Mater. Chem..

[31] Varaprasad K., Raghavendra G.M., Jayaramudu T., Yallapu M.M., Sadiku R. (2017). A mini review on hydrogels classification and recent developments in miscellaneous applications. J. Mater. Sci. Eng. C.

[32] Hanak B.W., Hsieh C.-y., Donaldson W., Browd S.R., Lau K.K.S., Shain W. (2018). Reduced cell attachment to poly(2-hydroxyethyl methacrylate)-coated ventricular catheters in vitro. J. Biomed. Mater. Res. Part B Appl. Biomater..

[33] Zainuddin Z., Barnard Z., Keen I., Hill D.J.T., Chirila V.T., Harkin D.G. (2008). PHEMA hydrogels modified through the grafting of phosphate groups by ATRP support the attachment and growth of human corneal epithelial cells. J. Biomater. Appl..

[34] Mei Y., Wu T., Xu C., Langenbach K.J., Elliott J.T., Vogt B.D., Beers K.L., Amis E.J., Washburn N.R. (2005). Tuning cell adhesion on gradient poly(2-hydroxyethyl methacrylate)-grafted surfaces. Langmuir.

[35] Deng J., Ren T., Zhu J., Mao Z., Gao C. (2014). Adsorption of plasma proteins and fibronectin on poly(hydroxylethyl methacrylate) brushes of different thickness and their relationship with adhesion and migration of vascular smooth muscle cells. Regen. Biomater..

[36] Ren T., Mao Z., Guo J., Gao C. (2013). Directional migration of vascular smooth muscle cells guided by a molecule weight gradient of poly(2-hydroxyethyl methacrylate) brushes. Langmuir.

[37] Minett T.W., Tighe B.J., Lydon M.J., Rees D.A. (1984). Requirements for cell spreading on polyHEMA coated culture substrates. Cell Biol. Int. Rep..

[38] Ravi M., Paramesh V., Kaviya S.R., Anuradha E., Solomon F.D.P. (2015). 3D Cell Culture Systems: Advantages and Applications. J. Cell. Physiol..

[39] Tibbitt M.W., Anseth K.S. (2009). Hydrogels as extracellular matrix mimics for 3D cell culture. Biotechnol. Bioeng..

[40] Aubin H., Nichol J.W., Hutson C.B., Bae H., Sieminski A.L., Cropek D.M., Akhyari P., Khademhosseini A. (2010). Directed 3D cell alignment and elongation in microengineered hydrogels. Biomaterials.

[41] Paterson S.M., Shadforth A.M.A., Shaw J.A., Brown D.H., Chirila V.T., Baker V.M. (2013). Improving the cellular invasion into PHEMA sponges by incorporation of the RGD peptide ligand: The use of copolymerization as a means to functionalize PHEMA sponges. J. Mater. Sci. Eng. C.

[42] Charrier E.E., Pogoda K., Wells R.G., Janmey P.A. (2018). Control of cell morphology and differentiation by substrates with independently tunable elasticity and viscous dissipation. Nat. Commun..

[43] Thermo Fisher S. (2012). Crosslinkers Technical Handbook.

[44] Argraves W.S., Suzuki S., Arai H., Thompson K., Pierschbacher M.D., Ruoslahti E. (1987). Amino acid sequence of the human fibronectin receptor. J. Cell Biol..

[45] Koteliansky V.E., Glukhova M.A., Benjamin V.M., Smirnov V.N., Filimonov V.V., Zalite O.M., Venyaminov S.Y. (1981). A Study of the Structure of Fibronectin. Eur. J. Biochem..

[46] Ravasco J.M., Faustino H., Trindade A., Gois P.M.P. (2019). Bioconjugation with Maleimides: A Useful Tool for Chemical Biology. Chem. Eur. J..

[47] Dolan C., Drouet F., Ware D.C., Brothers P.J., Jin J., Brimble M.A., Williams D.E. (2016). A new high-capacity metal ion-complexing gel containing cyclen ligands. RSC Adv..

[48] Wang Y., Fan S., Zhong W., Zhou X., Li S. (2017). Development and Properties of Valine-Alanine based Antibody-Drug Conjugates with Monomethyl Auristatin E as the Potent Payload. Int. J. Mol. Sci..

[49] Achilias D.S., Siafaka P.I. (2017). Polymerization Kinetics of Poly(2-Hydroxyethyl Methacrylate) Hydrogels and Nanocomposite Materials. Processes.

[50] Menter P. (2000). Acrylamide Polymerization—A Practical Approach; tech note 1156.

[51] Chaudhuri O., Gu L., Klumpers D., Darnell M., Bencherif S.A., Weaver J.C., Huebsch N., Lee H.-p., Lippens E., Duda G.N. (2016). Hydrogels with tunable stress relaxation regulate stem cell fate and activity. Nat. Mater..

[52] Gutekunst S.B., Grabosch C., Kovalev A., Gorb S.N., Selhuber-Unkel C. (2014). Influence of the PDMS substrate stiffness on the adhesion of Acanthamoeba castellanii. Beilstein J. Nanotechnol..

[53] Meakin J.R., Hukins D.W.L., Aspden R.M., Imrie C.T. (2003). Rheological properties of poly(2-hydroxyethyl methacrylate) (pHEMA) as a function of water content and deformation frequency. J. Mater. Sci. Mater. Med..

[54] Shapiro J.M., Oyen M.L. (2013). Hydrogel Composite Materials for Tissue Engineering Scaffolds. JOM.

[55] Peppas N.A., Huang Y., Torres-Lugo M., Ward J.H., Zhang J. (2000). Physicochemical Foundations and Structural Design of Hydrogels in Medicine and Biology. Annu. Rev. Biomed. Eng..

[56] Discher D.E., Janmey P., Wang Y.-L. (2005). Tissue Cells Feel and Respond to the Stiffness of Their Substrate. Science.

[57] Seo E., Kumar S., Lee J., Jang J., Park J.H., Chang M.C., Kwon I., Lee J.S., Huh I.Y. (2017). Modified hydrogels based on poly(2-hydroxyethyl methacrylate) (pHEMA) with higher surface wettability and mechanical properties. Macromol. Res..

[58] Eschbach F.O., Huang S.J. (1994). Hydrophilic-Hydrophobic Binary Systems of Poly(2-hydroxyethyl methacrylate) and Polycaprolactone. Part I: Synthesis and Characterization. J. Bioact. Compat. Polym..

[59] Ulu A., Balcioglu S., Birhanli E., Sarimeseli A., Keskin R., Koytepe S., Ates B. (2018). Poly(2-hydroxyethyl methacrylate)/boric acid composite hydrogel as soft contact lens material: Thermal, optical, rheological, and enhanced antibacterial properties. J. Appl. Polym. Sci..

[60] Technical Committee ISO/TC 194 (Biological Evaluation of Medical Devices) Biological Evaluation of Medical Devices Part 5: Tests for Cytotoxicity (ISO Standard No. 10993-5), 2009 International Organization for Standardization Web Site. https://www.iso.org/standard/36406.html.

[61] Technical Committee ISO/TC 194 (Biological Evaluation of Medical Devices) Biological Evaluation of Medical Devices Part 1: Evaluation and Testing within a Risk Management Process (ISO Standard No. 10993-1), 2018 International Organization for Standardization Web Site. https://www.iso.org/standard/68936.html.

[62] Omidian H., Park K., Kandalam U., Rocca J.G. (2010). Swelling and Mechanical Properties of Modified HEMA-based Superporous Hydrogels. J. Bioact. Compat. Polym..

[63] Jhaveri S.J., Hynd M.R., Dowell-Mesfin N., Turner J.N., Shain W., Ober C.K. (2009). Release of Nerve Growth Factor from HEMA Hydrogel-Coated Substrates and Its Effect on the Differentiation of Neural Cells. Biomacromolecules.

[64] Badea A., McCracken J.M., Tillmaand E.G., Kandel M.E., Oraham A.W., Mevis M.B., Rubakhin S.S., Popescu G., Sweedler V.J., Nuzzo R.G. (2017). 3D-Printed pHEMA Materials for Topographical and Biochemical Modulation of Dorsal Root Ganglion Cell Response. ACS Appl. Mater. Interfaces.

[65] Cox E.A., Sastry S.K., Huttenlocher A. (2001). Integrin-mediated Adhesion Regulates Cell Polarity. Mol. Biol. Cell.

[66] Schindelin J., Arganda-Carreras I., Frise E., Kaynig V., Longair M., Pietzsch T., Preibisch S., Rueden C., Saalfeld S., Schmid B. (2012). Fiji: An open-source platform for biological-image analysis. Nat. Methods.

