# Supplementary material for: A Co-Polymerizable Linker for the Covalent Attachment of Fibronectin Makes pHEMA Hydrogels Cell-Adhesive
Source: Gels. 2022 Apr 21;8(5):258. doi: 10.3390/gels8050258 (PMC9140594; doi:10.3390/gels8050258)
Supplement: Supplementary file 1 [file gels-08-00258-s001.zip › gels-1617656-supplementary.pdf]

Supplementary Information

A Co-Polymerizable Linker for the Covalent Attachment of Fibronectin Makes pHEMA Hydrogels Cell-adhesive

Laura Schumacher[1,2], Katharina Siemsen[3], Clement Appiah[1,2], Sunil Rajput[4], Anne Heitmann[1,2], Christine Selhuber-Unkel[4,5] and Anne Staubitz[1,2]**

1 University of Bremen, Institute for Organic and Analytical Chemistry, Leobener Straße 7, D-28359 Bremen, Germany

2 University of Bremen, MAPEX Center for Materials and Processes, Bibliothekstr. 1, 28359 Bremen, Germany, E-mail: staubitz@uni-bremen.de, Tel: +49-421-218 63210

3 Kiel University, Institute for Materials Science, Biocompatible Nanomaterials, Kaiserstr. 2, D-24143 Kiel, Germany

4 Heidelberg University, Institute for Molecular Systems Engineering (IMSE), INF 253, D-69120 Heidelberg, E-mail: selhuber@uni-heidelberg.de, Tel: + 49-6221-544935

5 Max Planck School Matter to Life, Jahnstraße 29, D-69120 Heidelberg

Table of Contents

1 Abbreviations 3
2 Chemicals and Solvents 5
3 Analytical Equipment 9
4 Experimental Procedures 10
5 ^{1}H NMR and $^{13}C\{^{1}H\}$ NMR Spectra 14
6 ATR-IR Spectra 16
7 Synthesis Under Oxygen and Nitrogen Atmosphere 17
8 Stress Strain Curves 18
9 Cell Culture 19
10 References 20

1 Abbreviations

Table S1. List of abbreviations.

Abbrev.	Full name	Abbrev.	Full name
aq.	aqueous	GC	gas chromatography
CA	cycloaddition	GC-MS	gas chromatography and mass spectrometry
CC	column chromatography	HEMA	Hydroxyethylmethacrylate
d	doublet	HEPES	2-(4-(2-Hydroxyethyl)-1-piperazinyl)-ethansulfonsäure
DCM	dichloromethane	m	Multiplet (for ^{1}H-NMR)
DMF	dimethylformamide	m	*meta*
EGDMA	Ethylenglycoledimetharcylate	MA	methacrylic acid
EtOH	Ethanol	Me	methyl
FBS	fetal bovine serum	MeCN	Acetonitrile
FN	fibronectin	MeOH	methanol
MS	mass spectrometry	ppm	parts per million
MTT	3-(4,5-Dimethylthiazol-2-yl)-2,5-diphenyl tetrazoliumbromid	q	quartet
NHS	N-hydroxysuccinimide	quin	quintet
NMR	nuclear magnetic resonance	Ph	phenyl
o	ortho	Ref52wt	rat embryonic fibroblasts 52 wild type

p	para	r.t.	room temperature
PBS	phosphate-buffered saline	s	singlet
MS	mass spectrometry	sat.	saturated
MTT	3-(4,5-Dimethylthiazol-2-yl)-2,5-diphenyl tetrazoliumbromide	t	triplet
NHS	*N*-hydroxysuccinimide	TEMED	*N, N, N', N'*-Tetramethylethylendiamin
NMR	nuclear magnetic resonance	TLC	thin layer chromatography
o	ortho	THF	tetrahydrofurane
p	para	TMS	trimethylsilyl
PBS	phosphate-buffered saline	UV	ultraviolet
MS	mass spectrometry	Vis	visible
PEG	poly ethyleneglycol	APS	Ammoniumpersulfate
PEGX	poly ethyleneglycol with X ethylene units	MTT	3-(4,5-Dimethylthiazol-2-yl)-2,5-diphenyl tetrazoliumbromid

2 Chemicals and Solvents

All chemicals and solvents were commercially available and were used as received unless noted otherwise. The dry solvents were either purchased or dried with a solvent purification system (SPS), from Inert Innovative Technology, Inc. Company. Inert reactions were carried out using Schlenk techniques under a dry, inert nitrogen atmosphere. The copper catalyst was stored in the glove box form the Inert, Innovative Technology, Inc. Company (0.1 ppm O_2 and 0.1 ppm H_2O).

If not noted otherwise, all reagents were used as received.

Table S2. List of suppliers and purity of the used solvents.

**Solvent**	**Supplier / Drying Agent**	**Purity**
Acetonitrile	ChemSolute	99,9 %
Chloroform-d_1	Deutero	100% D
Dichloromethane	Merck	≥ 99.9 % HPLC grade, dry from SPS
Dimethyl sulfoxide	VWR	HPLC grade, dry from SPS
N,*N*-Dimethylformamide	Acros Organics	99.5%
Ethanol	Fischer Chemicals	> 99.8%
Ethyl acetate	Fischer	≥ 99.9 %
n-Hexane	VWR	98 %
n-Pentane	Sigma Aldrich	95%
Toluene	Merck	99.7 %

Table S3. List of suppliers and purity of the used chemicals.

Reagent	Supplier	Purity	Comments
Acetic anhydride	Acros Organics	97 %	
Ammonium persulfate	Sigma Aldrich	99.9 %	
β-Alanine	Alfa Aesar	98 %	
Dicyclohexylcarbodiimide	Sigma Aldrich	99 %	
Diethylene glycol	JK Chemicals	99 %	
Ethylene glycol dimetharcylate	Sigma Aldrich	95 %	Stab. with MEHQ
Hydroxyethyl methacrylate	TCI	95 %	Stab. with MEHQ
Hydroxylamine hydrocholoride	Acros Organics	97 %	
Hydroxysuccinimde	Apollo Scientific		
Maleic anhydride	Roth	≥ 99.5 %	
Methacrylic acid	TCI	> 99 %	Stab. with MEHQ
4-Methoxyphenole	Sigma Aldrich	≥ 98 %	
2-Propanol	Walter CMP	Chem. rein	
p-TSA	Sigma Aldrich<br>Abcr	98.5 %<br>97 %	
2-Sulfobutanedioic acid	Sigma Aldrich	70 %	in H_2O

N, *N*, *N*', *N*'-Tetramethylethylendiamin	Acros Organics	99,5 %	Purified by redestillation
Triethylamine anhydrous	Fluorochem	99%	anhydros
1, 3, 5 – Methoxybenzene	Sigma Aldrich	≥ 99 %	

Table S4. List of suppliers and purity of the used chemicals for cell cultivation and staining.

Reagent	Supplier	Purity	Comments
Accutase	-		
Calcein-AM	BD science		
Dulbecco's MEM	Merck		
Fluorobrite DMEM	Gibco		Sterile
GlutaMAX	Gibco		
HEPES	Sigma Aldrich	≥ 99.5 %	
Hoechst-	ThermoFischer		
MEM Earle's	Merck		Without $NaHCO_3$, L-Glutamine and Phenol red
3-(4,5-Dimethylthiazol-2-yl)-2,5-diphenyl-tetrazoliumbromide	Sigma Aldrich	98 %	
Penicilin Streptomycin	SigmaAldrich	-	10 mg/mL streptomycin
Propidiumiodid	ThermoFischer	-	AlexaFlour 647
Trypsin/EDTA solution	Merck	0.5 %/ 0.2 % (w/v)	In PBS

3 Analytical Equipment

Column chromatography was performed on silica gel 60 obtained from Merck with a pore size of 15 – 40 µm. Analytical thin layer chromatography (TLC) was performed on silica gel 60 F_{254} plates from Merck. Visualization was achieved by a $KMnO_4$ (0.0475 M) containing staining solution.

^{1}H NMR spectra and ^{13}C{^{1}H} NMR spectra were recorded on a on a Bruker DRX-600 (600 MHz) spectrometer at 298 K. Chemical shifts are referenced to the residual proton of the deuterated solvent ($CDCl_3$: δ (^{1}H) = 7.26 ppm, δ (^{13}C{^{1}H} = 77.2 ppm). All chemical δ shifts are given in parts per million (ppm) and all coupling constants *J* in Hz. Full assignment of the peaks was achieved with the aid of 2D NMR techniques (^{1}H/1H COSY and ^{1}H/^{13}C HSQC).

Electron Impact (EI) ionization mass spectra were obtained on the double focusing mass spectrometer MAT 95+ or MAT 8200 from Finnigan Mat. Samples were measured by direct inlet or indirect inlet method with a source temperature of 200 °C. The ionization energy of the electron impact ionization was 70 eV. All signals were reported with the quotient from mass to charge *m/z*. High resolution mass spectrometric (HRMS) measurements were performed in the positive ion mode using a JEOL-Accu TOF 4GGCV EI mass spectrometer or on the double focusing mass spectrometer MAT 95+ or Mat 8200 form Finnigan Mat. Electron ionization (EI) was performed using an ionization potential of 70 eV. Atmospheric pressure chemical ionization (APCI) experiments were performed on a Bruker Impact II from Bruker Daltonics. The calculated isotopic distribution for each ion was in agreement with experimental values.

IR spectra were recorded on a Nicolet Thermo iS10 Scientific IR spectrometer with a diamond-ATR-unit. The resolution was 4 cm^{-1}. Relative intensities of the IR bands were described by s = strong, m = medium or w = weak.

4 Experimental Procedures

Synthesis of 3-maleimidopropionic acid *N*-hydroxysuccinimide ester

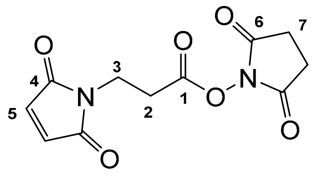

In a 250 mL flask β-alanine (5.00 g, 56.1 mmol) was added in one portion to the already stirring solution of maleic anhydride (5.50 g, 56.1 mmol) in dry DMF (50 mL) under a N_2-atmosphere. The mixture was stirred for 5 h at 23 °C. Subsequently, it was cooled to 0 °C and hydroxysuccinimide (7.90 g, 70.0 mmol) was added, followed by the addition of dicyclohexyl carbodiimide (23.1 g, 112 mmol) and additional amount of DMF (50 mL). The reaction mixture was allowed to warm up to 23 °C and was stirred for 18 h.

The reaction mixture was filtered to remove the dicyclohexylurea and rinsed with DMF (15 mL). The filtrate was concentrated under reduced pressure (7 mbar, 45 °C). Traces of DMF were removed by co-evaporation with octane fractions (total 110 mL). The residue was partially dissolved in DCM (150 mL); the remaining precipitate was filtered to give the pure product as a first fraction. The DCM filtrate was washed with a sat. aqueous solution of $NaHCO_3$ (3 x 150 mL). The organic phase was collected and washed once with brine (150 mL). The organic phase was separated and dried with $MgSO_4$, filtered and evaporated under reduced pressure. The solid residue was then suspended in cold ethyl acetate (80 mL) and the suspension was centrifuged. The solid was kept, and after combining it with the product precipitate from the first wash, the solid was dried under vacuum to yield the product as a colorless powder (8.00 g, 30.0 mmol, 54 %).

^{1}H NMR (600 MHz, $CDCl_3$): δ = 6.75 (s, 2H, H-5), 3.94 (t, *J* = 6.90 Hz, 2H, H-3), 3.03 (t, *J* = 6.90 Hz, 2H, H-2), 2.83 (s, 4H, H 7) ppm.

^{13}C NMR (600 MHz, $CDCl_3$): δ = 170 (C-4), 196 (C-6), 166 (C-1), 135 (C-5), 33.3 (C-3), 30.1 (C 2), 25.9 (C-7) ppm.

Melting point, T = 168 °C

HRMS (ESI) *m/z* (%): found 267.06213 [M+H]$^+$, calcd. for [M+H]$^+$ $C_{11}H_{11}N_2O_6$ 267.06171, found 289.03216 [M+Na]$^+$, calcd. for [M+Na]$^+$ $C_{11}H_{10}N_2NaO_6$ 289.04366.

IR (ATR): $\tilde{\upsilon}$ = 3480 (w), 3120 (w), 2980 (s), 2850 (s), 1840 (m), 1710 (m), 1465 (s), 1397 (s), 1310 (m), 1200 (s), 1050 (m), 1095 (s), 915 (m), 815 (m), 695 (s), 650 (m). cm^{-1}.

The NMR information were in agreement with the results in the given literature.[1]

Synthesis of 2-(2-hydroxyethoxy)ethyl methacrylate[2]

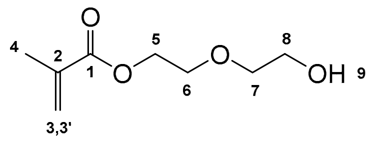

A 500 mL two-neck round bottom flask equipped with a Dean-Stark apparatus and a condenser was filled with para-toluenesulfonic acid monohydrate (810 mg, 4.71 mmol, 0.100 eq.), toluene (300 mL), diethylene glycol (20.0 g, 188 mmol, 4.00 eq.) and methoxyphenol (1.17 g, 9.40 mmol, 0.2 mol%) as inhibitor. The mixture was heated at reflux for 2 h to remove water. Subsequently, methacrylic acid (4.06 g, 47.1 mmol, 1.00 eq.) was added and the mixture stirred at reflux for another 3 h. The solvent was removed under reduced pressure and the residue was purified by flash chromatography on silica gel (hexane/EtOAc: 3/2 to 100 % EtOAc) to afford the product as a colourless oil.

For long term storage a few crystals of p-methoxyphenol were added.

^{1}H NMR (600 MHz, $CDCl_3$): δ = 6.12 (s, 1H, H-3), 5.57 (s, 1H, H-3'), 4.30 (m, 1H, H-5), 3.75 (m, 1H, H 8), 3.73 (m, 1H, H-6), 3.60 (m, 1H, H-7), 1.94 (s, 1H, H-4) ppm.

^{13}C NMR (600 MHz, $CDCl_3$): δ = 167 (C-1), 136 (C-2), 126 (C-3), 72.0 (C-6), 68.7 (C-7), 64.3 (C-5), 60.7 (C-8), 18.5 (C-4) ppm.

HRMS (ESI) *m/z* (%): found 197.07829 $[M+Na]^+$, calcd. for $[M+Na]^+$ $C_8H_{14}NaO_4$ 197.07980.

IR (ATR): $\tilde{\upsilon}$ = 3427 (m), 2874 (m), 2250 (m), 1712 (s), 1637 (m), 1453 (s), 1377 (w), 1377 (w), 1319 (s), 1297 (s), 1169 (s), 1129 (s), 1066 (m), 907 (s), 950 (s), 815 (m), 727 (s). cm^{-1}.

The NMR information were in agreement with the results in the given literature.[2]

Synthesis of 3-maleimidopropionic acid diethyleneglycol methacrylate

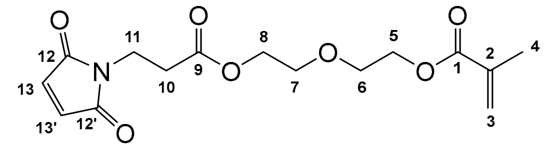

2-(2-Hydroxyethoxy)ethyl methacrylate (1.26 g, 7.20 mmol, 1.10 eq.) was dissolved in ethyl acetate (6 mL) and triethylamine (2.60 mL, 19.7 mmol, 3.00 eq.) was added. 3-Maleimidopropionic acid-*N*-hydroxysuccinimide ester (1.75 g, 6.58 mmol, 1.00 eq.) was subsequently added and the reaction mixture (suspension) was heated at reflux for 3 h. The solvent of the orange suspension was evaporated under reduced pressure. Purification of the crude product by column chromatography gave **3** as an orange oil which was stored at 0 °C (945 mg, 2.91 mmol, 44%).

$\mathbf{R}_f$ = 0.71 (ethyl acetate / *n*-hexane, 3 / 2);

^{1}H NMR (600 MHz, $CDCl_3$): δ = 6.70 (s, 2H, H-13, H-13a), 6.13 (s, 1H, H-4a), 5.58 (s, 1H, H-4b), 4.31-4.29 (m, 2H, H-5), 4.25-4.21 (m, 2H, H-8), 3.83 (t, $^3J_{10,11}$ = 7.1 Hz, 2H, H-11), 3.75-3.72 (m, 2H, H-6), 3.70-3.69 (m, 2H, H-7), 2.67 (t, $^3J_{10,11}$ = 7.1 Hz, 2H, H-10), 1.65-1.41 (m, 3H, H-3) ppm;

^{13}**C NMR** (600 MHz, $CDCl_3$): δ = 170.7 (C-9), 170.3 (C-12, 12a), 167 (C-3), 136 (C-2), 134 (C-13, 13a), 125 (C-4), 69.1 (C-7), 69.0 (C-6), 63.8 (C-8), 63.7 (C-5), 33.6 (C-11), 32.8 (C-10), 18.3 (C-1) ppm;

HRMS (ESI) *m/z* (%): found 326.12398 $[M+H]^+$; 348.10537 $[M+Na]^+$; 364.07931 $[M+K]^+$, calcd. for $[M+H]^+$ $C_{15}H_{20}NO_7$ 326.12398; calcd. for $[M+Na]^+$ $C_{15}H_{19}NO_7Na$ 348.10592; calcd. for $[M+Na]^+$ $C_{15}H_{19}NO_7K$ 364.07986.

IR (ATR): $\tilde{\upsilon}$ = 3475 (w), 3110 (w), 2956 (m), 1703 (s), 1636 (m), 1512 (m), 1445 (m), 1406 (m), 1377 (w), 1318 (m), 1296 (s), 1260 (m), 1168 (s), 1129 (s), 1075 (w), 1042 (m), 950 (s), 827 (s), 723 (w), 695 (s) cm^{-1}.

5 ^{1}H NMR and ^{13}C{^{1}H} NMR Spectra

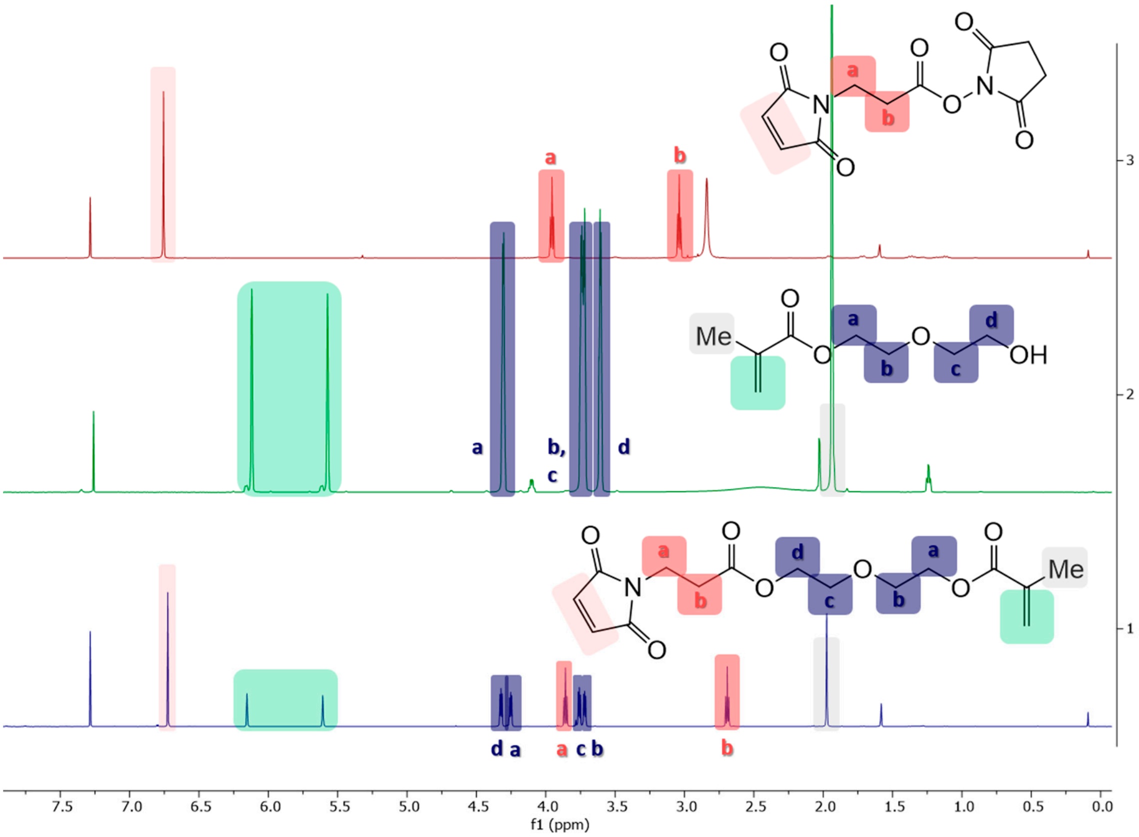

Figure S1. Comparison of ^{1}H NMR spectra of the starting materials (3-maleimidopropionic acid *N*-hydroxysuccinimide ester and 2-(2-hydroxyethoxy)ethyl methacrylate) and the bio-linker (3-maleimidopropionic acid diethyleneglycol methacrylate).

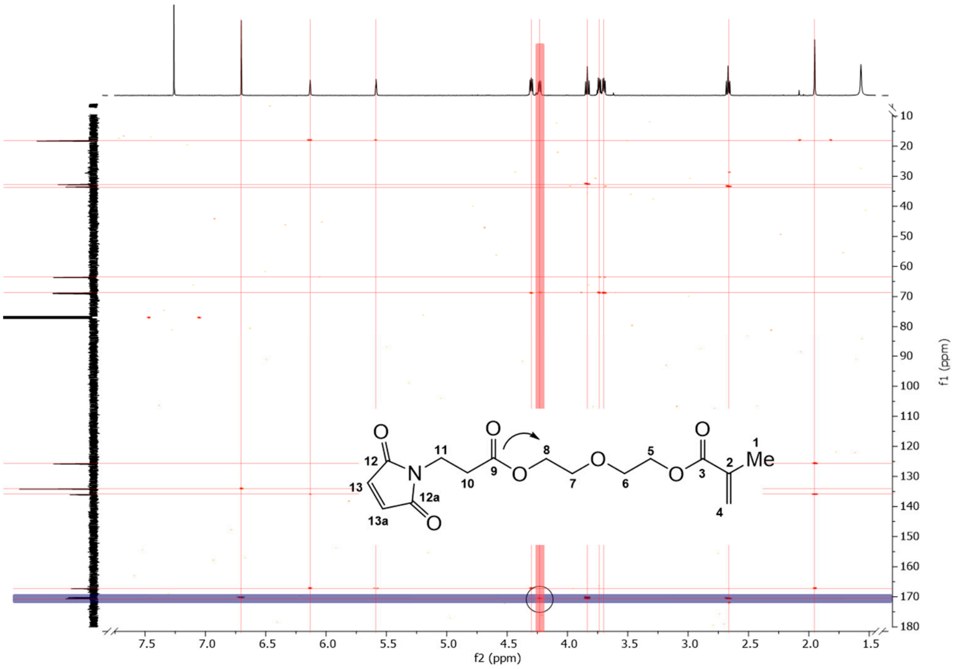

Figure S2. HMBC spectra of the bio-linker (3-maleimidopropionic acid diethyleneglycol methacrylate). The coupling between C9 and H8 is indicated with an arrow and marked in the spectrum, to proof the success of the esterification reaction.

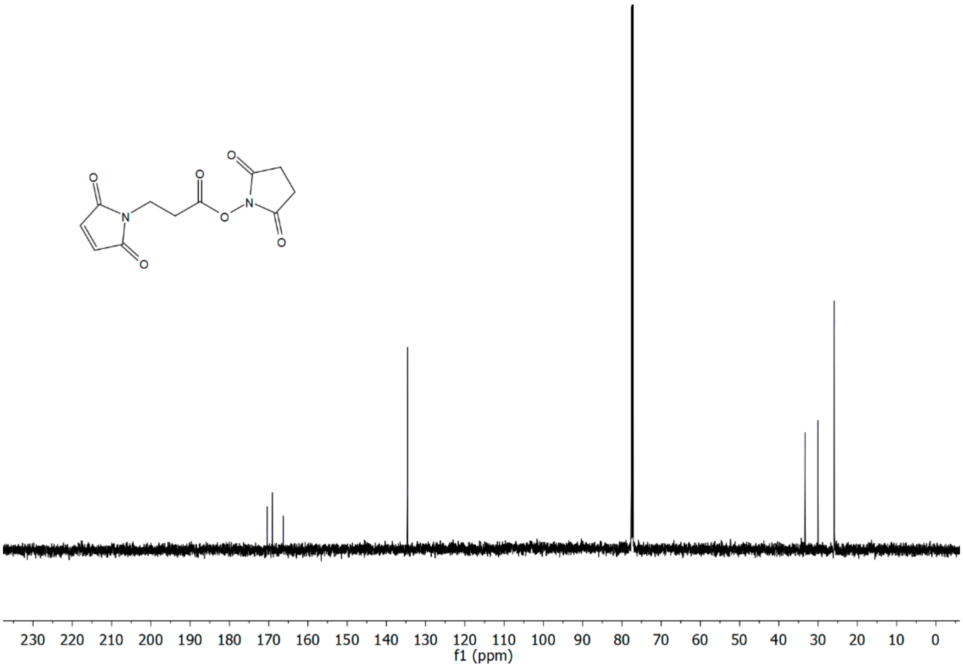

Figure S3. $^{13}C\{^{1}H\}$ NMR spectrum of the starting material (3-maleimidopropionic acid *N*-hydroxysuccinimide ester).

6 ATR-IR Spectra

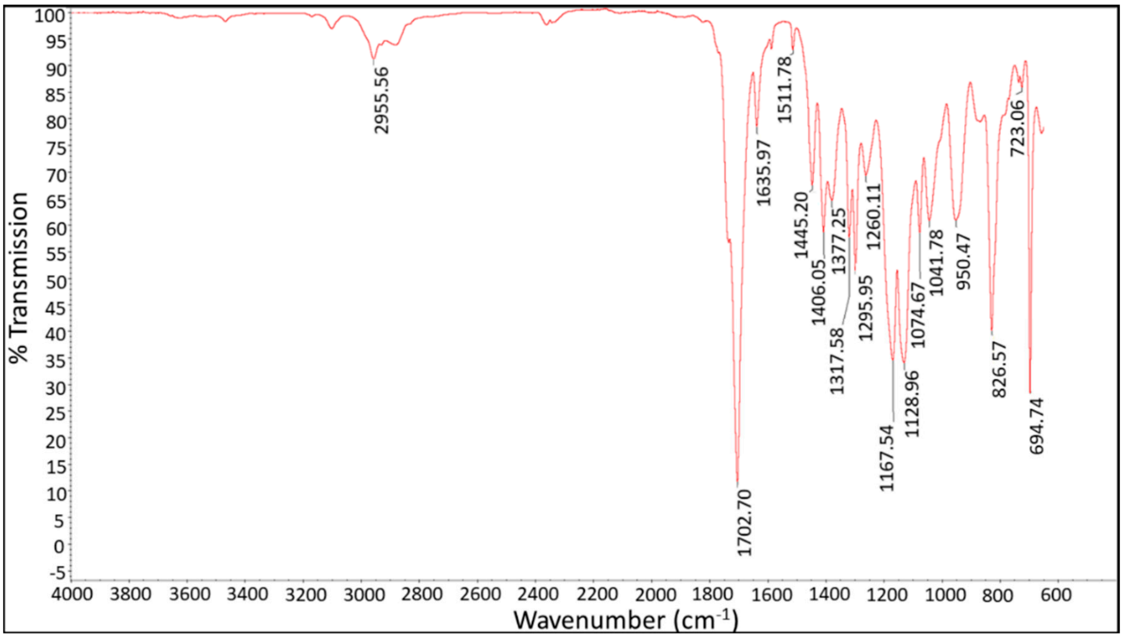

Figure S4. ATR-IR spectrum of the bio-linker (3-maleimidopropionic acid diethyleneglycol methacrylate).

7 Synthesis Under Oxygen and Nitrogen Atmosphere

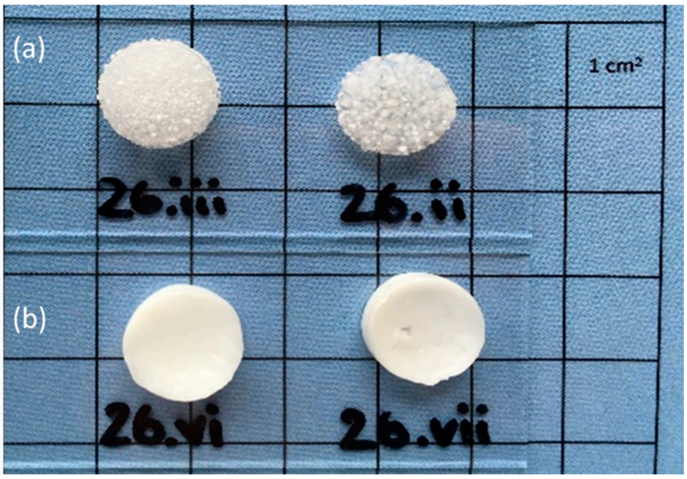

Figure S5. (a) hydrogels produced under oxygen atmosphere at 80 °C (b) hydrogels produced under nitrogen atmosphere at 80 °C.

8 Stress Strain Curves

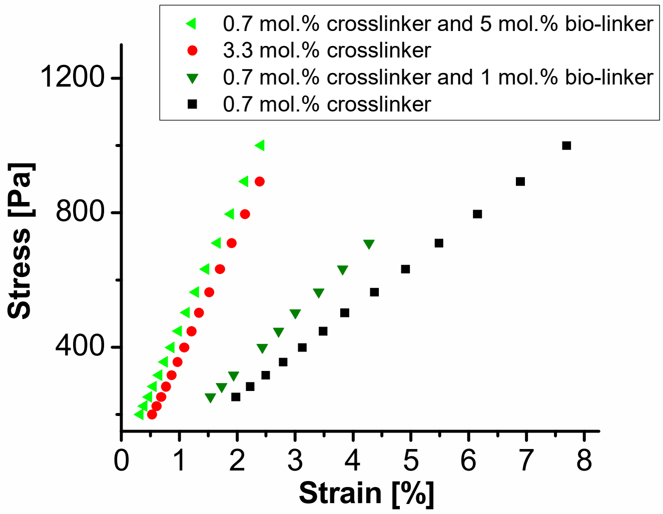

Figure S6. Stress-strain curves of the synthesized hydrogels with different percentages of cross-linker and bio-linker.

9 Cell Culture

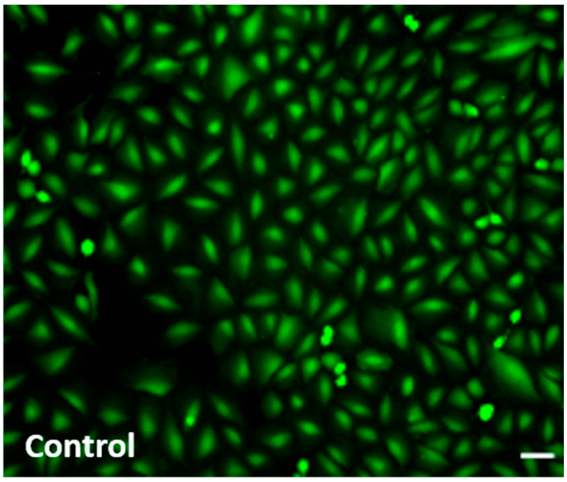

Figure S7. Control of Ref 52 wt after 24h of incubation on a 12-well plate (Sarstedt).

10 References

(1) Dolan C.; Drouet F.; Ware D. C.; Brothers P. J.; Jin J.; Brimble M. A. Williams D. E. A new high-capacity metal ion-complexing gel containing cyclen ligands. *RSC Adv.* **2016,** *6,* 23645–23652, DOI: 10.1039/c6ra00604c.

(2) Wang Y.; Fan S.; Zhong W.; Zhou X.; Li S. Development and Properties of Valine-Alanine based Antibody-Drug Conjugates with Monomethyl Auristatin E as the Potent Payload. *Int. J. Mol. Sci.* **2017**, *18*, 1860, DOI: 10.3390/ijms18091860.
